# Synthesis and Gas-Sensing Property of Highly Self-assembled Tungsten Oxide Nanosheets

**DOI:** 10.3389/fchem.2018.00452

**Published:** 2018-10-05

**Authors:** Liangbin Hu, Pengfei Hu, Yong Chen, Zehui Lin, Changjun Qiu

**Affiliations:** School of Mechanical Engineering, University of South China, Hengyang, China

**Keywords:** crystal structure, defects, nanosheets, highly self-assembled, gas-sensitivity

## Abstract

We report the synthesis of tungsten oxide (WO_3_) nanosheets using a simple yet efficient hydrothermal technique free of surfactantand template. The WO_3_ nano-sheets are self-assembled as well to form ordered one-dimensional chain nanostructure. A comprehensive microscopic characterization reveals that the nano-sheets have triangular and circular two different shape edges, dislocation and stacking faults are also observed, which should have implications for our understanding of catalytic activity of ceria. We also propose a growth mechanism for the nano-sheets. As a result of this unique morphology, this WO_3_ nano-sheets are found to show excellent gas-sensing properties which can use as promising sensor materials detecting ethanol with low concentration.

## Introduction

Tungsten trioxide WO_3_ nanomaterials are extensively applied in electrochromic device, gas sensor and photocatalysts filed (Hai et al., 2016; Zhang et al., 2018). Great effort has been devoted to the control the specific size and shape of WO_3_ nanoparticles which can significantly impact properties of materials. Up to now, the WO_3_ nanoparticles with avariety of morphologies (such as nano-wires, nano-rods, nano-plate, nano-spheres and so on) have been syhthesized successfully via high-intensity ultrasound, rapid microwave, hydrothermal synthesis and other methods (Chai et al., 2016, Hu et al., 2017a, Xu et al., 2017; Chen et al., 2018; Parthibavarman et al., 2018; Zhan et al., 2018). Among all of those applied methods, hydrothermal synthesis technique has been concerned due to the merits of simple operation, low energy consumption, the possibility for large-scale industrialization and so on.

Here, we report the WO_3_ nano-sheets with highly self-assembled architecture synthesized via the hydrothermal synthesis process. Defects are observed in the nano-sheets, which may play a key role in affecting properties of WO_3_ nano-sheets. In addtion, we also test the response and selectivity of the sensor fabricated from the WO_3_ nano-sheets.

## Materials and methods

All the reagents were analytical grade and without further purification. We adopted a facile hydrothermal method to synthesize the nanostructures. First of all, 32 ml of deionized water and 8 ml glycerol (C_3_H_8_O_3_) were mixed into a mixture, then 1.6 nmol of sodium tungstate dihydrate (Na_2_WO_4_·2H_2_O) and 3 nmol oleic acid (C_18_H_34_O_2_) were dispersed into the mixture, and stirred for 15 min with a magnetic stirrer. Secondly, the pH of the mixture was adjusted to 1.25 by HCl. After stirring for 15 min, the solution was transferred into the Teflon-lined stainless steel autoclave and treated at 120°C during 12 h under autogenously pressure. Finally, the obtained particles were washed by deionized and alcohol to remove the unexpected ions by high-speed centrifugation and then dried at 60°C for 10 h in air.

The characterization of speciemen as our previous work (Chen et al., 2013, 2014; Hu et al., 2017b). The process of measuring the gas sensitivity of the prepared nanomaterials is described in the literature (Guo and Wang, 2016). Response of the sensors was defined as the ratio of Ra (resistances in air) to Rg (resistances in target gases).

## Results

**Figure 1:**
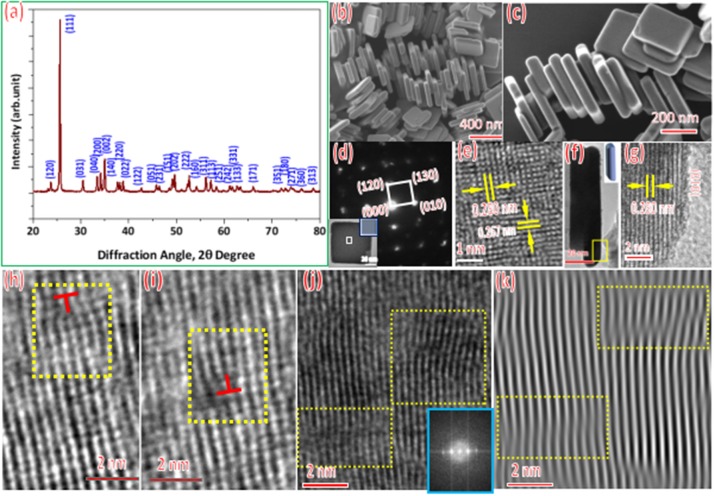
Characterization of the samples prepared by hydrothermal method:**(a)** XRD spectrum, **(b–c)** SEM images, **(d–g)**TEM images, insert of **(d)** shows the TEM image of a WO3 nanoparticle, insert of **(f)** shows the model of the side of the nanosheet, **(h–k)** defects of samples, insert of **(j)** shows the Fast Fourier transform of the HRTEM image.

Figure 1a shows a typical XRD spectrum of the products, the diffraction peaks match well with those of a standard WO_3_·H_2_O with orthorhombic structure (JCPDS No. 84-0886). The WO_3_·H_2_O nano-particles exhibit rectangle shape with an average size of ~400 nm and thickness of 30nm (Figure 1b). In addtion, one-dimensional chain nanostructure is self-assembled by the quadrilateral faces on the both sides of the nano-sheets. The face of nano-sheet are flat (Figures 1b,c). The corresponding SADP identifies that the structure of WO_3_ nano-sheets is orthorhombic (Figures 1d). Figure 1e shows a high-resolution TEM (HRTEM) image taken around the corner of a nano-sheet. We determined the lattice spacing of the perpendicularlattice fringes to be ~0.280 and ~0.267 nm which are belong to the WO_3_ (010) and (120) planes respectively. Figure 1g shows a HRTEM image taken from the corner area of the nano-sheet Figure 1f from which lattice spacing is determined to be ~0.280 nm, in line with the distance between {010} planes of WO_3_. In addtion, the edges of the nano-sheets are in an arc shape (insert of Figure 1f). Two different direction edge dislocations: positive edge dislocation (Figure 1h) and negative edge dislocation (Figure 1i) are deteced by the HR-TEM, which should be critical for the properties of WO_3_. Apart from the defects on the surface, we also observed the stacking faults in nano-sheet (Figure 1j), which should impact the properties of WO_3_ nanoparticles as well. The stacking fault was observed obviously by the one-dimensionally filtered HR-TEM images of the WO_3_ nano-sheet (Figure 1k). Those dislocations and stacking faults may affect the catalytic activity or other properties of nano material (Wang et al., 2011, 2014, 2016; Sun et al., 2015).

**Figure 2:**
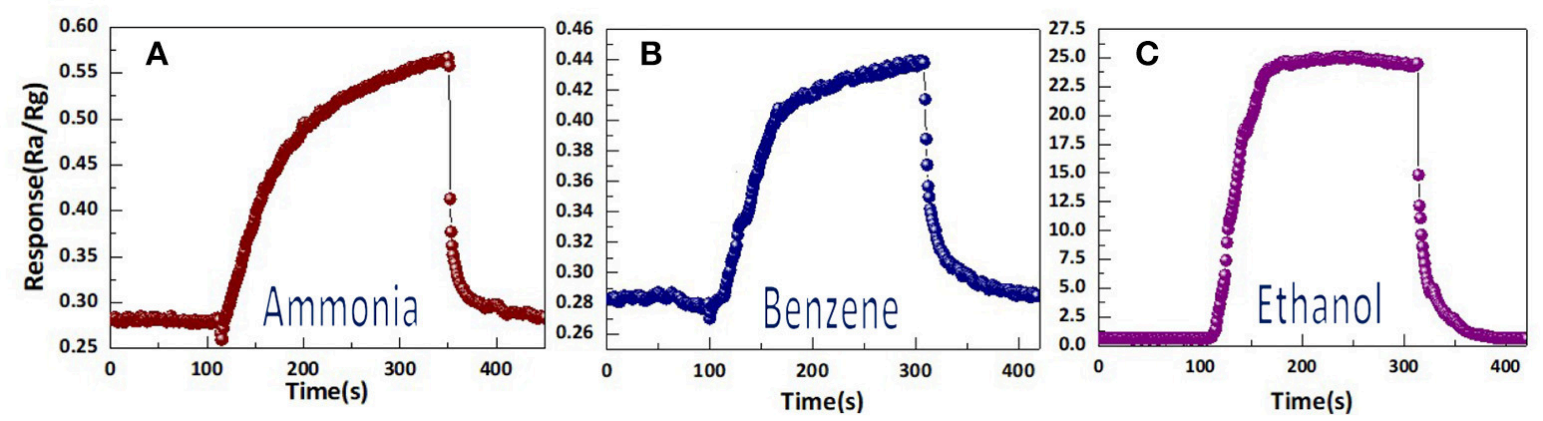
The response of highly self-assembled tungsten oxide nanosheets sensor toward 30 ppm of different testing gases: **(a)** ammonia, **(b)** benzene, **(c)** ethanol.

Figure 2 shows the gases (NH_3_, CH_3_OH, C_6_H_6_) response of the sensor based on WO_3_ nanosheets. All of the gases were tested at an operating temperature of 300°C with a concentration of 30 ppm. In Figure 2, the results indicate that the sensor exhibited little responses to NH_3_, C_6_H_6_, to indicated that it was insensitive to NH_3_, C_6_H_6_. For ethanol, the highest response of the sensor was 25.6, while the responses to NH_3_ and C_6_H_6_ were no >1.

## Discussion

In light of the aforementioned microstructural characterization, we propose a likely growth mechanism for the nanosheet. First, the Na_2_WO_4_·2H_2_O is ionized to ${\text{WO}}_{4}^{-}$. Then, the ${\text{WO}}_{4}^{-}$ ion react with H^+^ which ionized by HCl, forming the H_2_WO_4_ suspension. The H_2_WO_4_ suspension decomposes, at the high temperature and pressure during hydrothermal process, resulting in to the nucleation of WO_3_. The oleic acid acts as a soft template and controls the growth rate of different crystal plane owing to its selective absorption and desorption behavior. Then most of WO_3_ nano-sheets with {010} exposure planes are self-assembled, forming one-dimensional chain nanostructures, due to the addition of oleic acid. The formation process can be described as follows:

(1) $$\begin{array}{r} {\text{Na}}_{2}{\text{WO}}_{4}\cdot 2{\text{H}}_{2}\text{O}\to 2\text{N}{\text{a}}^{+}+{\text{WO}}_{4}^{-}+{2\text{H}}_{2}\text{O} \end{array}$$

(2) $$\begin{array}{r} \text{HCl}\to {\text{H}}^{+}+{\text{Cl}}^{-} \end{array}$$

(3) $$\begin{array}{r} {2\text{H}}^{+}+{\text{WO}}_{4}^{-}\to {\text{H}}_{2}{\text{WO}}_{4} \end{array}$$

(4) $$\begin{array}{r} {\text{H}}_{2}{\text{WO}}_{4}\to {\text{WO}}_{3}+{\text{H}}_{2} \end{array}$$

We imply that the WO_3_ nano-sheets can act as an efficient gas-sensing material for selective detection of ethanol. Such the sensing performance due to the fact that the diffusion of ethanol and its oxidation with O^−^ or O^2−^ are very rapidly in nanoplates structures (Xiao et al., 2017). When sensor prepared by the WO_3_ nanosheets is exposed in air, the resistance of the WO_3_ nanosheets is increased by oxygen molecules which adsorbed on the surfaces of the WO_3_ nanosheets, trapping electrons in the conduction band and forming oxygen species (O^−^, O^2−^). As ethanol is introduced to the sensor, the oxygen species (O^−^, O^2−^) react with ethanol molecules on the surface of the WO_3_ nanosheets, which will release trapped electrons to conduction band and resistance of the WO_3_ nanosheets is decreased (Ahmad et al., 2013).

## Conclusions

We have adopted the hydrothermal technique to synthesize highly self-assembled WO_3_ nano-sheets using the tungsten resource Na_2_WO_4_·2H_2_O and the soft template oleic acid. We demonstrated that the WO_3_ nano-sheets are mainly exposed with {010} planes and crystal defects such as edge dislocations and stacking faults exist in single crystalline nano WO_3_ by microscopic investigations, which may be important for the catalytic activity of WO_3_. We indicate that the WO_3_ nano-sheets could be used as promising sensor material for detecting CH_3_OH with low concentration.

## Author contributions

LH synthesized and characterized the microstructure of highly self-assembled tungsten oxide nanosheets and wrote the manuscript. PH and ZL tested the response and selectivity of the sensor fabricated from the WO_3_ nano-sheets. YC and CQ directed the experiment. All authors read and approved the manuscript.

### Conflict of interest statement

The authors declare that the research was conducted in the absence of any commercial or financial relationships that could be construed as a potential conflict of interest.
